# Toward Sustainable Polyurethane Foams: Effects of Corn Cob Fibers and Silver Nanoparticles on Mechanical Properties and Antimicrobial Activity

**DOI:** 10.1021/acsomega.4c07118

**Published:** 2024-11-22

**Authors:** Gabriel Vinicius Alves Silva, Gabriel Fornazaro, Gabriel Vinicius Inacio Benati, Mychelle Vianna Pereira Companhoni, Francielle Pelegrin Garcia, Jean Halison de Oliveira, Eduardo Radovanovic, Silvia Luciana Fávaro

**Affiliations:** † Department of Mechanical Engineering, State University of Maringá, Colombo 5790, 87020-900 Maringá, PR, Brazil; ‡ Institute of Physics, Federal University of Mato Grosso do Sul, Av. Costa e Silva, s/no, 79.070-900 Campo Grande, MS, Brazil; § Department of Basic Health Science, State University of Maringá, Colombo 5790, 87020-900 Maringá, PR, Brazil; ∥ Department of Chemistry, State University of Maringá, Colombo 5790, 87020-900 Maringá, PR, Brazil

## Abstract

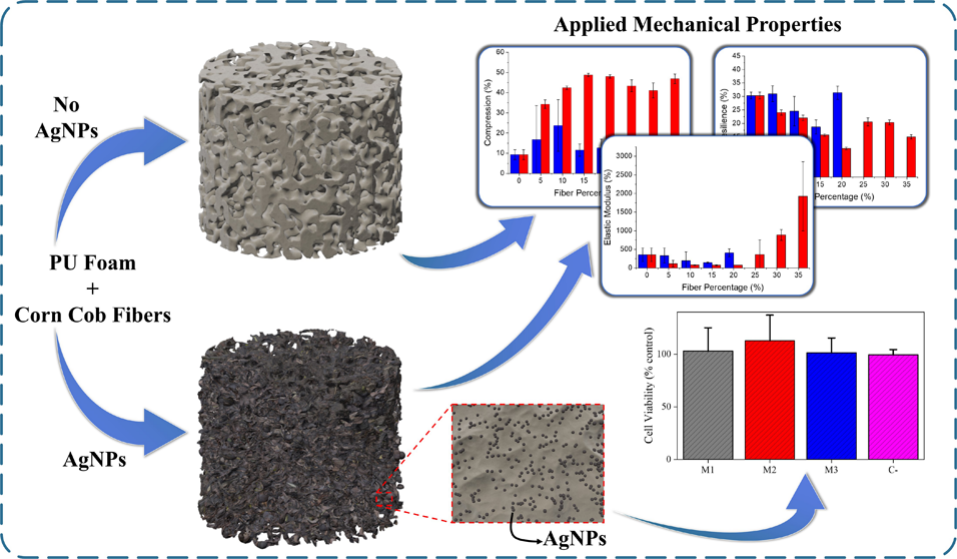

Polyurethane foams (PFs) are widely used in mattresses, upholstery, and insulation, but disposal is difficult due to the disintegration time and environmental hazards of synthetic polyol. This work investigates a sustainable alternative by replacing poly(ethylene glycol) (PEG) with corn cob fibers and incorporating antibacterial silver nanoparticles (AgNPs). Corn cob fibers and sodium hydroxide-treated fibers were used to make foams, with corn cob fibers substituting PEG at 5–30 wt %. In terms of durability and elastic modulus, low-fiber-content foams matched nonfiber counterparts. Higher fiber content (more than 20%) resulted in divergent properties with potential benefits. In terms of viscoelastic qualities, foams with a 15% fiber content outperformed nonfiber foam. Antimicrobial testing revealed that AgNP-infused foams with 15% corn cob fibers effectively inhibited microbiological growth.

## 1 Introduction

The development of environmentally friendly and commercially viable products based on natural resources, applied to matrices and reinforcements, has attracted the interest of many researchers in recent years and is in continuous growth.^1−3^ Polyurethanes, a diverse group of polymers, are widely used in various products, such as coatings, adhesives, shoe soles, mattresses, and foam insulation. However, the basic chemistry of each type is essentially the same.^4^

Among the polyurethane products, flexible polyurethane foams (PF) are three-dimensional polymers supported by cross-links. Moreover, they are flexible materials with open cells, are air-permeable, reversible to deformation, and can be produced by prioritizing different properties such as modulus of elasticity, hardness, and resilience so that no other material provides such properties.^5^ Numerous researchers have recently investigated using natural fibers as reinforcement in composite materials.^6−8^ Adding natural fibers into polyurethane matrices can modify mechanical strength, reduce costs, and enhance the material’s biodegradability.^9,10^

Natural fibers of plant origin are primarily derived from leaves, stems, or seeds and consist of cellulose molecules combined with other substances such as pigments, resins, gums, fats, and waxes, often considered impurities.^11^ Brazil cultivates a variety of natural fibers, including sisal, coconut, jute, ramie, curauá, sugar cane bagasse, soy, rice husks, and other grains. Among these fibers, corn cob stands out as a renewable and abundant material with potential as a reinforcement in polymer composites. Corn cob fibers are characterized by high strength, stiffness, and low weight, making them valuable biodegradable resource. Furthermore, corn cob fibers are nontoxic, cost-effective, facilitate efficient processing, and are widely available, making them suitable for various industrial applications.^12^ The field of natural fiber applications has been expanding, including applications in the textile, automotive, civil, and furniture industries used as reinforcement in thermosetting and thermoplastic polymer matrices. Recently, natural fibers have shown promise as absorbent materials for heavy metals in industrial waste treatment.

Silver nanoparticles (AgNPs) have been widely used across various industries due to their excellent antimicrobial, antifouling, deodorizing, hypoallergenic, electrical conductivity, and self-cleaning properties.^13^ Silver products have strong inhibitory and bactericidal effects against a broad spectrum of infections. AgNPs exhibit antifungal, anti-inflammatory, and antiviral activities, among others.^14^ According to Su-juan Yu, most AgNPs are dissolved and washed away upon first contact with water, suggesting minimal impact on the degradation of materials incorporating AgNPs.^15^ As presented by Keley Zhu, the increasing use of AgNPs potentially causes marine ecosystem changes; however, the environmental impacts of man-made AgNPs are still poorly studied.^16^

In this work, we investigate the effects of replacing polyethylene glycol (PEG) with corn cob fibers and incorporating silver nanoparticles (AgNPs) in polyurethane foams (PFs) for the development of a sustainable and antimicrobial material, with focus on foam properties, microbial growth inhibition, and cytotoxicity.

## 2 Experimental Section

### 2.1 Materials

4,4′-Methylene diphenyl diisocyanate (MDI), tin octanoate 95 wt %, poly(dimethylsiloxane), glycerol, and polyethylene glycol (PEG; mol wt 1500; 75 mg KOH/g) were purchased from Sigma-Aldrich Co. and used as received. The corn cobs were acquired from a farmer in Maringá, Paraná, Brazil.

### 2.2 Fiber Treatment Procedure

For the fiber treatment, the corn cobs were cleaned, dried in an oven at 60 °C for 48 h, ground using a knife mill, and sieved (∼355 μm) for further modification. The mercerization step was adapted from ref (17), where the fibers were soaked in caustic soda at different concentrations and times. Next, the corn cob fibers were treated in NaOH 10 wt % for 30 min at room temperature, then washed with deionized water until they reached a pH of ca. 7, and dried at 60 °C for 24 h. The silver nanoparticles (AgNPs) deposition was assembled directly in the fiber surface procedure by heating 30 mL of deionized water, adding 0.5 g of the fibers and 0.335 g of silver nitrate, and stirring by reflux for 1 h. After that, the treated fibers (TF) were filtered, washed with deionized water, and air-dried.

For the foam preparation, the fibers *in natura* (FN) and treated fibers (TF) were added in different concentrations during the synthesis, as shown in Table 1. The foam that exhibited the best results in the mechanical tests was developed by using fibers with silver nanoparticles (AgNPs) for comparison. The fibers were added in PEG and one droplet of glycerin, and then the mixture was added to 3 g of MDI and 4 drops of tin octanoate. Vigorous stirring was performed until foam formation. As shown in Table 1, the fiber exchange was carried out on PEG, increasing by 5% increments, with the highest fiber content of 35%. Finally, 24 h after the synthesis, the variance was analyzed to investigate the statistical influence of the variables in the foam synthesis.

**Table 1:** Synthesis of the Foams

fiber composition (%)	fiber (g)	PEG (g)	MDI (g)	PMHS (mL)
0	0.0	6.0	3.0	0.6
5	0.5	5.5	3.0	0.6
10	1.0	5.0	3.0	0.6
15	1.5	4.5	3.0	0.6
20	2.0	4.0	3.0	0.6
25	2.5	3.5	3.0	0.6
30	3.0	3.0	3.0	0.6
35	3.5	2.5	3.0	0.6

### 2.3 Scanning Electron Microscopy

The morphological characterization of the fibers was performed by scanning electron microscopy (SEM) on an FEI Quanta 250 microscope. The foam composites were observed by SEM and energy-dispersive X-ray spectroscopy (EDS) on an FEI Scios Dual-beam. The samples were sputtered with a gold layer for 120 s at 60 A for the analyses.

### 2.4 Fourier Transform Infrared Spectroscopy

The chemical modification and stability of the fibers were carried out using an infrared spectrophotometer attached with an attenuated total reflectance accessory (FTIR-ATR, Digilab Scimitar Series Pike Miracle ATR) operating from 4000 to 400 cm^–1^ with a resolution of 4 cm^–1^ and 64 scans.

### 2.5 Resilience Test

The resilience of the foams was assessed through the ball rebound test method, as per the ASTM D3574,^18^ employing a custom-made resiliometer. The experimental setup involved the release of a ball within a graduated tube, which subsequently affected the foam material. The resilience of the foam was quantified by measuring the highest position reached by the ball in the tube without contacting its walls.

### 2.6 Elastic Modulus

The elastic modulus of the foams was tested based on the ASTM D3574.^18^ The procedures were performed in a Stable Micro Systems TA.XTplus Texture Analyzer.

### 2.7 Compression Set Test

The mechanical durability of the foams was measured according to the ASTM D395-B.^19^ The test was performed by adding foam between two metallic plates to evaluate the permanent deformation.

### 2.8 Ash Content

The ash content was evaluated according to ASTM D5630.^20^ The foams were previously cut and weighed. In some way, its mass was not less than 2 g. The samples were placed in a ceramic vessel that had already been weighed. Samples were put in an oven at 900 °C for 15 min. A ceramic vessel with ashes from foam had the mass noted; the difference is the ashes.

### 2.9 Antimicrobial Activity

The microorganisms used for the antimicrobial evaluation were *Escherichia coli* (ATCC 25922), *Pseudomonas aeruginosa* (ATCC 27853), and *Staphylococcus aureus* (ATCC 25923), cultivated in *Mueller Hinton Broth* (MHB)(Difco) at 37 °C. Further, the antifungal activity was performed using *Candida albicans* (ATCC 10231), cultivated in *Sabouraud Dextrose Broth* (SDB)(Difco) at 37 °C. Before each experiment, microorganisms were cultivated in the respective broths at 37 °C for 24 h. Then, cell density was adjusted using sterile 0.9% saline tubes to achieve turbidity identical to the McFarland scale of 0.5, corresponding to 1 × 10^8^ CFU/mL for bacteria and 1 × 10^6^ CFU/mL for yeast.

### 2.10 Agar Diffusion Assay

The foam samples were cut into small cylinders with a diameter of 6 mm and then sterilized by using ethylene oxide. To perform a disk-diffusion test, a sterile cotton swab was dipped into the standardized suspension of each microorganism, and the excess fluid was removed by pressing the swab against the tube wall. Then, the microorganisms were seeded on the surface of the culture media (MH agar for bacteria or SD agar for yeast). Finally, the samples were placed on the surface of the agar plates and incubated at 37 °C for 24 h. The diameters of the growth inhibition zones were determined with modifications.^21^

### 2.11 Broth Microdilution Assay

Both fibers (FN and FAG) and the milled foams (PF, PF with fiber *in natura* (PFN) and PF with fibers/AgNPs (PFAG)) were evaluated in order to verify their antimicrobial potential.

To determine the Minimum inhibitory concentrations (MICs) and Minimum bactericidal/fungicidal concentrations (MBC/MFC), all of the samples were evaluated by the microdilution assay using MHB or SDB according to the Clinical and Laboratory Standards Institute.^22^ Briefly, 100 μL of broth samples were distributed in each well of 96-well plates. Then, 100 μL of each powder was added in the first well (initial concentration, 1 mg/mL), proceeding with serial dilution. Inocula were then standardized as described above and diluted 1:10 (bacteria) or 1:100 (yeast). Finally, 5 μL of the inoculum was added to each well of the plate. Inoculum control (wells without fiber) and negative control (wells without fibers and inocula) were also performed. The plates were incubated at 37 °C, and the MIC was recorded after incubation for bacteria for 24 and 48 h for yeast. The MIC was defined as the lowest concentration of compounds at which the microorganism tested did not demonstrate visible growth. Further, the MBC and MFC concentrations were evaluated using the subculture technique on Muller-Hinton Agar or Sabouraud Agar for bacteria and yeast, respectively. For this, 10 μL was removed from each well (MIC concentration, one below and one above) and transferred to the agar, which was then incubated at 37 °C for an additional 24 h.

Additionally, the samples (FAG, PF, PFN, and PFAG) were evaluated following the same procedure with adaptations. For this, 10 mg/mL of each sample was incubated in a 24-well microplate (950 μL of the respective culture medium and 50 μL of the standardized microorganism/well) at 37 °C for 24 h. The inoculum control (no sample, only inoculum) and the sample control (no inoculum, only the sample) were also performed. After the incubation, the wells were observed to verify each material’s inhibitory potential (no visible microorganism growth).

### 2.12 Cytotoxicity Assay

Fibroblast cell line L929 (clone NCTC 929, L CELL, L929; ATCC CCL1, Manassas) was maintained in DMEM supplemented with 10% fetal bovine serum at 37 °C in a 5% CO_2_ environment. PBS and trypsin/EDTA were used to subculture the cells before they reached confluence. To investigate the cytotoxicity activity of FAG, 2.5 × 10^5^ cells per well were plated in 96-well plates. After 24 h incubation, different fiber concentrations with the nanoparticle (10, 5.0, 2.5, 1.0, and 0.5 mg/mL) were added to the 96-well plate in duplicate in two independent experiments followed by incubation for 24 h. Negative control was performed (cells in the medium with no material).

Moreover, to evaluate all samples of milled foams (PF, PFN, and PFAG), 10 mg/mL of each sample was added to the wells plated with 2.5 × 10^5^ cells per well in 24-well plates in duplicate in two independent experiments. At the end of the incubation period (24 h), the cell viability was evaluated by the MTT method. Briefly, an MTT solution at a concentration of 2 mg/mL was added per well, and the plate was incubated for 4 h at 37 °C with 5% CO_2_ (in the dark). Subsequently, DMSO was added, and the reading was performed in a microplate reader (BIO-TEK Power Wave XS) at 570 nm. Then, by nonlinear regression, the % of cell viability was calculated.

## 3 Results and Discussion

### 3.1 FTIR Analysis

**Figure 1:**
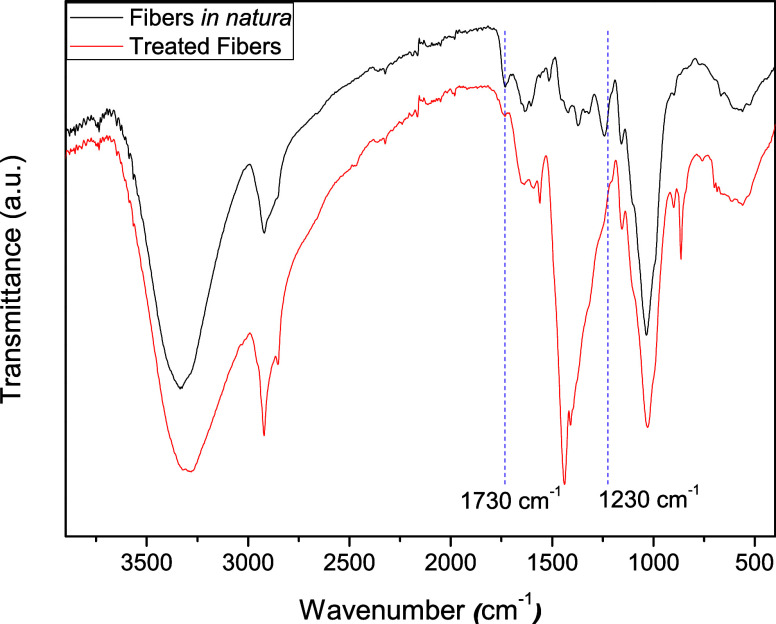
FTIR spectrum analyses of treated and corn cob fibers *in natura*.

The corn cob fibers were treated to remove impurities and expose hydroxyl groups, increasing the fiber reactivity and facilitating their interaction with isocyanate. The surface modification of the treated fibers is exposed in Figure 1. The peaks at 1730 and 1230 cm^–1^ for treated fibers *in natura* are attributed to C=O and C–O stretching due to lignin and hemicellulose groups in the fiber structure. The band reduction after the treatment improves the diisocyanate interactions in the fibers.^23−25^

### 3.2 Scanning Electron Microscopy

**Figure 2:**
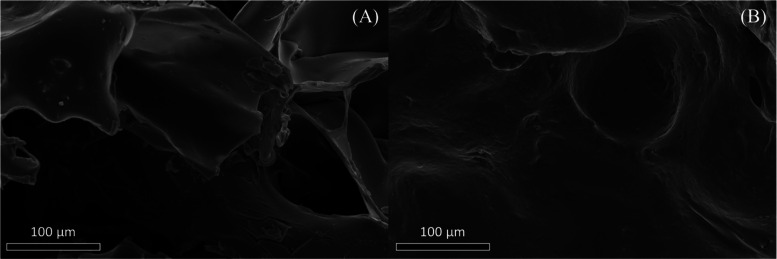
SEM micrographs of the *in natura* (A) and treated (B) corn cob fibers.

The scanning electron micrographs of the corn cob fibers reveal changes in the surface structure after treatment (Figure 2). The debris distributed under the fiber is a set of impurities and metabolic waste from the plant.^22^ The treated fibers (Figure 2B) had a clean surface induced by the lignin and hemicellulose removal corroborating with the FTIR results.^26,27^

### 3.3 Mechanical Properties of the Foams

**Figure 3:**
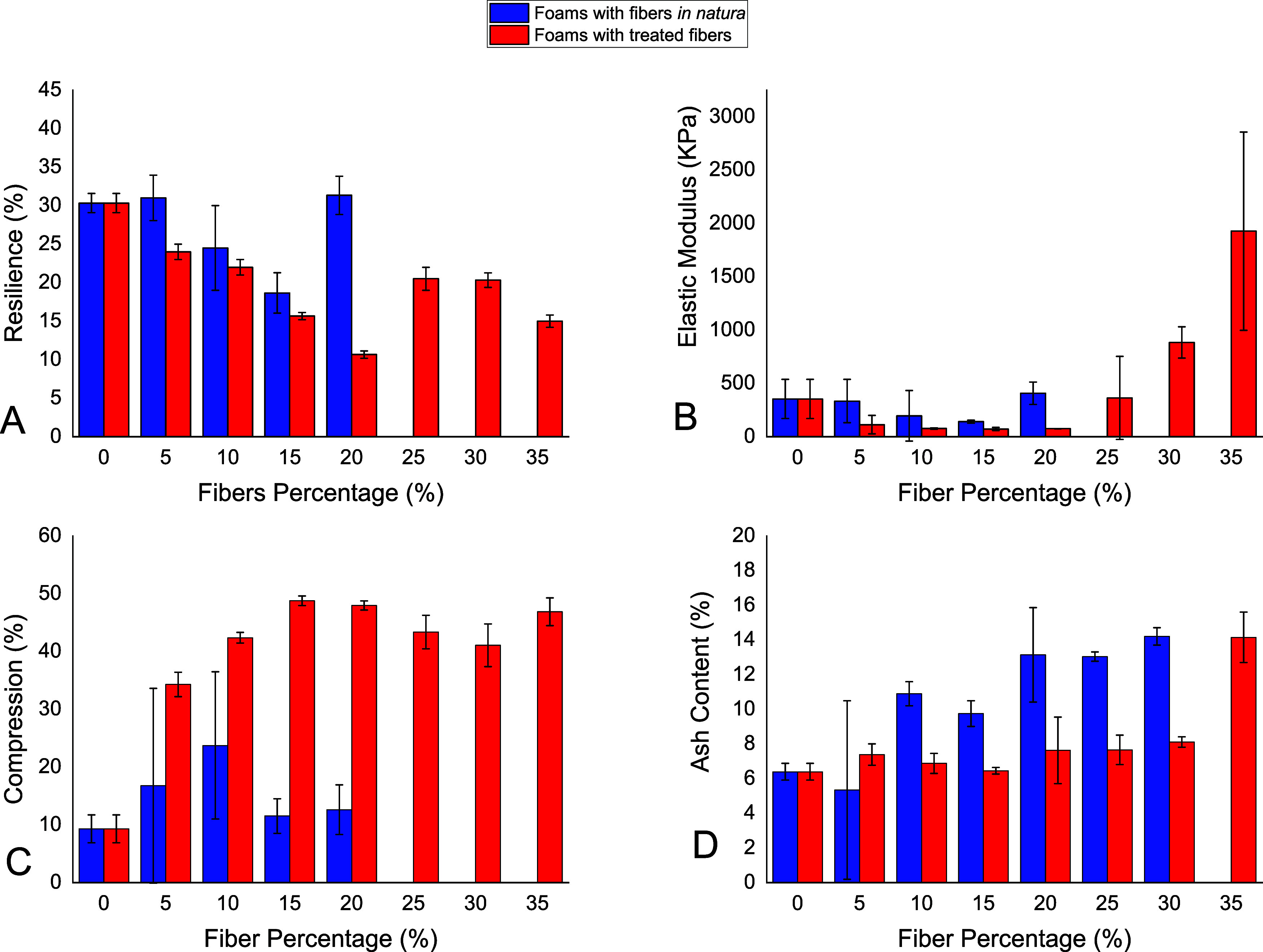
Mechanical tests and ash content for the composites. (A) Resilience tests, (B) elastic modulus, (C) compression set, and (D) ash content evaluation.

The polyol was changed for fibers until the maximum quantity produced foam. This point reached 20 wt % for the polyurethane foams using fibers *in natura* (PFN) and 35 wt % using treated fibers (PF-TF). Foams using 10 wt % fewer fibers maintain the characteristics of the raw PF, and 15 and 20 wt % presented a viscoelastic behavior where the compressive relaxation is delayed until it reaches the initial form.^24^ In comparison, foams with over 20 wt % of fibers showed more toughness due to the higher quantity of fibers. The mechanical properties and ash content evaluation are shown in Figure 3. First, in the resilience tests (Figure 3A), the fiber content improves the cross-linked bonds between the fiber and isocyanate, decreasing the resilience and reducing the foam elasticity, absorbing less energy.^28^ The lower resilience values intensify for the PF-TF due to the fragility in the fiber structure in the mercerization step, discussed later in the compression session.^29^ The nonlinear characteristic for the viscoelastic foams (15 and 20 wt % of fibers) occurs due to a delay after the compressive interaction, presented in Figure 3A,B.

The resilience and elastic modulus are related properties as observed that when resilience decreases, the stiffness increases; consequently, the elastic modulus increases.^3^ Furthermore, knowing that larger resilience values give better absorption of the impact energy. At the same time, the elastic modulus confers softness, and the objective was to prepare a composite with an intermediate of both characteristics. Foams with high fiber content exhibited a larger elastic modulus, unlike the PFN (Figure 3B). The foams using 5 and 10 wt % of the fibers *in natura* presented elastic modulus and resilience close to the PFN, inducing a maximum rate of fibers to maintain the properties of the foams with no substitution.

The incorporation of corn cob fibers, whether in nature or treated, altered the reactive environment within the foam system due to the surplus hydroxyl groups provided by the fibers. This led to significant changes in the foam’s stoichiometric balance, particularly with the hydroxyl and isocyanate groups. The treated fibers with more exposed hydroxyl groups further amplified this effect, as observed through FTIR analysis. Consequently, as fiber content increased, the mechanical properties shifted, with reduced resilience and elastic modulus at higher fiber concentrations. Despite these alterations, foams with fiber contents up to 15% retained the desired properties for use in flexible applications.

The change in stoichiometric ratio may have contributed to the decreased resilience and elastic modulus in foams with higher fiber content (>20%). However, these results suggest that foams with high fiber loading may be more suitable for thermal and acoustic insulation applications, where higher rigidity and compression resistance are desirable. For future investigations, a more precise adjustment of the hydroxyl/isocyanate ratio, considering the contribution of both treated and untreated fibers, to optimize mechanical properties without compromising the biodegradability and sustainability of the material.

The compression set test was realized for a long-time compression evaluation of the foams (Figure 3C). The PFN exhibited lower compression resistance as the resilience decreased. On the other hand, the PF-TFs presented a higher permanent compression rate independent of the fiber content. Chang and co-workers^30^ reported the influence of the NaOH concentration in composites reinforced with natural fibers, where the composites with an enhanced permanent compression were obtained using 2 wt % or superior NaOH concentrations.^31^

The PF-TFs provided an ash content of 7 wt % (Figure 3D), as the PFNs gave higher values, indicating that removing impurities, lignin, and hemicellulose caused by the chemical treatment decreased the ash content. The fiber content also enhances the ash content once the fibers are organic.

### 3.4 Tukey’s Test

Three sets of samples were evaluated for the Tukey test: PFNs, PF-TFs, and polyurethane foams without fibers (PF). According to Tukey’s test, the mechanical properties data related to the foams are significant since this property gives the applications for the composite. Furthermore, for the elastic modulus test, the difference was insignificant except for the foams with 30 and 35 wt % of fibers. The more significant difference from that of PFN was resilience, as previously discussed. For the compression set values, there was a difference represented by an enhancement of compression rather than a loss.

Hence, the PF using 15 wt % of fibers was selected to incorporate the silver nanoparticles (AgNPs), as they presented lower variability in mechanical tests, lower permanent deformation, and a viscoelastic profile.

### 3.5 Fibers with AgNPs

**Figure 4:**
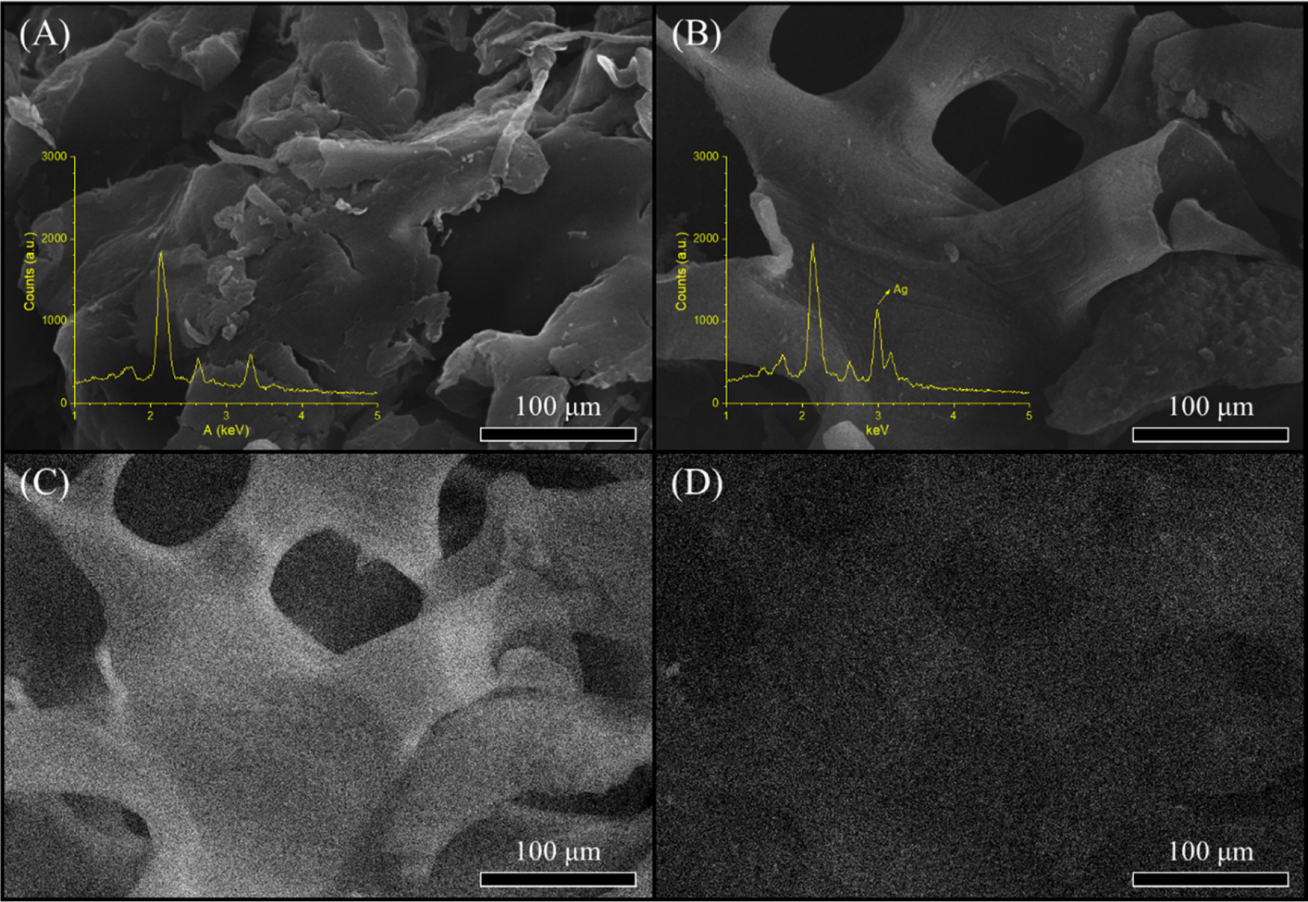
Scanning electron micrographs and energy-dispersive X-ray spectra of the corn cob fibers *in natura* (A), treated with AgNPs (B), mapping for carbon (C) and silver (D).

The AgNPs synthesis was visually confirmed as the solution became yellow since the silver ions (Ag^+^) were reduced to Ag^0^. SEM images (Figure 4) show the AgNPs depositing over the fiber surface. The silver reduction was conducted by the hydroxyl groups present in the cellulose structure.^32^ The EDX analysis exhibited a peak for silver close to 3 keV for the treated composite, indicating the deposition of the AgNPs in the surface of the fiber as observed in the inset graph of Figure 4B.^33^ These results complement the micrographs of the fibers where the images of the treated fibers present AgNPs all over the surface, different from the fibers *in natura* (Figure 4A).

To evaluate the AgNPs over the fiber surface, EDS mapping was made for carbon (Figure 4C) and silver (Figure 4D). Carbon covers all of the fiber surfaces as a constituent element of the material. However, Figure 4D presents no agglomerated silver above the fiber, giving a homogeneous layer making the material complete for the biological tests.

### 3.6 Foams with AgNPs

**Figure 5:**
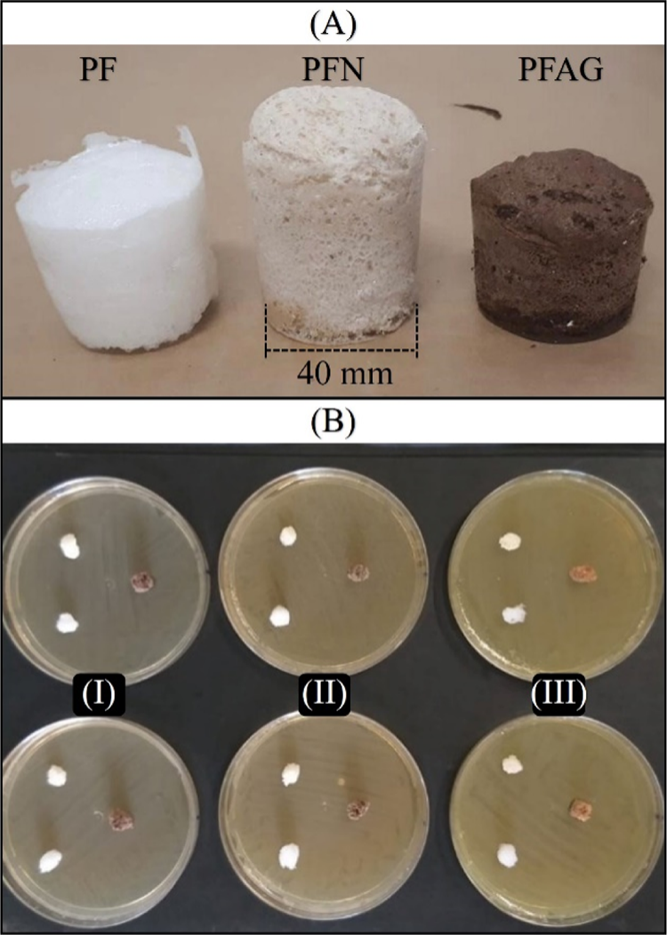
(A) Color change comparison after the treatment steps and (B) agar diffusion assay of samples PF, PFN, and PFAG against (I) *S. aureus*, (II) *E. coli*, and (III) *P. aeruginosa*.

The color change also occurred in the foams due to the presence of fibers. Figure 5A compares the PFs from left to right: foam without fibers, foam with 15% fibers in natura, foam with 15% fiber, and AgNPs deposition.

Mechanical tests were performed to verify the mechanical properties of the foams with adding AgNPs compared to those with fibers *in natura*. Table 2 presents the resilience and the permanent compression tests.

**Table 2:** Results of Resilience and Permanent Compression Tests in Foams with Fibers Modified by AgNPs Deposition

foam composition	resilience (%)	permanent compression (%)
15% FN	18.67 ± 3.21	11.53 ± 2.99
15% FAg	21.67 ± 0.58	5.89 ± 3.53

There was a slight change in the resilience of foams with fibers modified by AgNPs deposition.^34^ However, it is worth noting that adding these nanoparticles improved the performance of the foams in the permanent compression test. Although it deviates from the foam with fiber *in natura*, the change in property was positive, allowing the foam to withstand prolonged use compared to the foam with fiber *in natura*.

### 3.7 Antimicrobial Assays

The agar diffusion assay was performed on PF, PFN, and PFAG. It was expected to obtain the formation of an inhibition halo around the foam with nanoparticles because of the antimicrobial action of AgNPs.^35^ However, as observed in Figure 5B, no halo formation was observed; that is, the foam with the active agent did not show antimicrobial activity by this method.^2^ One of the factors for this result may be the structure of the polymeric matrix, imprisoning the fibers with silver nanoparticles, thus preventing them from interacting with microorganisms. The lack of inhibition in the assay on agar plates can be explained by the fact that no free silver ions are available, suggesting that the foam sample may not show toxicity. Toxicity is directly associated with Ag^+^, which can modify biological molecules, cells, and human organs.^36^

As the foams did not show inhibition in the agar method, the serial microdilution assay in broth was performed for PFAG. We aimed to determine the antimicrobial property of the AgNPs. The determined MIC and MBC/MFC are shown in Table 3. For *S. aureus* and *P. aeruginosa* bacteria, the silver nanoparticle fiber only presented a microbiostatic action, while for the other microorganisms, it also showed a microbicidal action.

**Table 3:** Antimicrobial Activity of the FAG by Broth Microdilution

nicroorganisms	MIC (mg/mL)	MBC (mg/mL)	MFC (mg/mL)
*S. aureus*	0.312	>5	NA
*E. coli*	0.625	1.25	NA
*P. aeruginosa*	0.625	>5	NA
*C. albicans*	0.312	NA	1.25

The tests with the milled foams (concentration of 10 mg/mL) in the 24-well plate showed antimicrobial activity since the PFAG inhibited the growth of the microorganisms compared to the negative control (no treatment). At the same time, PF and PFN presented the growth of microorganisms (data not shown).

After this result, the microbicidal action of the milled foams was further analyzed. The results evidenced the microbicidal action of the fiber against all tested microorganisms at the concentration of 10 mg/mL, which was expected regarding the results of Table 3.^37^ Meanwhile, FAG had a microbicidal action for *E. coli* and *C. albicans* and a microbiostatic action against *P. aeruginosa*. and *S. aureus*. Thus, it only inhibited the growth of microorganisms. In their work, Santos et al.,^38^ using polymeric blends with AgNPs, the microbicidal action is perceived against *E. coli*, *P. aeruginosa*, and *S. aureus*. In addition, the blends with AgNPs were less cytotoxic than the other samples, with only the polymers.^39^ In the present work, AgNPs in the fiber and foam allowed bacteriostatic action for the material, suggesting that its use for the desired applications meets the expected requirements. In this way, this new material, the fibers with nanoparticles, can be used in several applications, showing that the technology using corn cob fibers processed by the present route and impregnated with AgNPs has the potential for use in the industry through the processing of a residue solid, adding value to industrial waste.

### 3.8 Cell Viability

**Figure 6:**
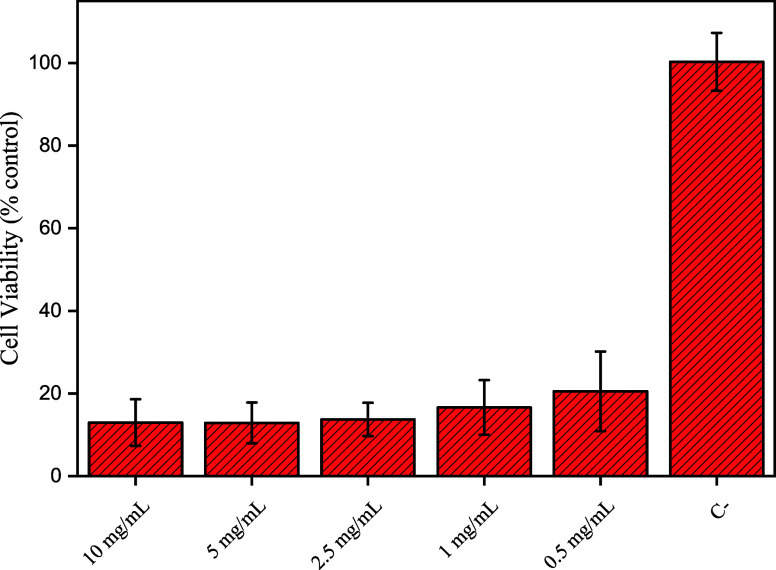
MTT cellular viability of FAG at different concentrations.

The viability tests are listed in Figure 6. The cell viability in the presence of FAG sample in the lowest concentration tested was lower than 25%, which classifies this sample as cytotoxic, as ISO 10993-5 defines a cytotoxic sample with cellular viability lower than 80%, and it offers health risks. The main reason for lower viability is the small size of the nanoparticles deposited on the fiber.^40^ Sometimes viability can be over 100%, that is indicated that the material evaluated promotes cell proliferation.^41−43^ However, it is crucial to consider that other nanoparticle characteristics can interfere with toxicity, such as form, chemical composition, and surface charge.^44^

The results of cell viability of PFAG in the concentration of 10 mg/mL showed 80% of living cells, so these composites would not be harmful to the user’s health and protect the product from microbiological challenges.

In this work, the AgNPs entrapped in the fiber in the PU matrix allowed the bacteriostatic action, suggesting their application to the desired requirements. So, the corn cob fibers and AgNPs, the newly investigated material, can be employed in many applications, showing that the processing of corn cob fiber technology processed through the present route and impregnated with AgNPs has potential use in industry, due to solid residue processing, adding value to industrial waste.

As the external organ of the body, the skin is the first and foremost barrier to protecting the body against external microbes.^45^ So, the products in contact should present antimicrobial characteristics; however, no toxicity is required. Therefore, cytotoxicity assays were done to determine if the fiber with AgNPs and PU composite with fibers and AgNPs showed any risk to the health. Some recent studies showed the AgNPs in PU foams as biomaterials for wound healing.^46^

**Figure 7:**
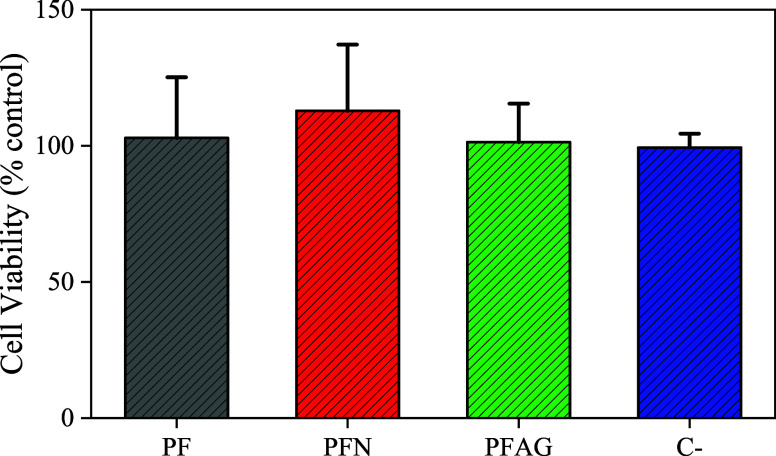
L929 cell viability of PFAG determined by the MTT method.

The result of the present investigation can be observed in Figure 7, which showed that there was no toxicity to the fibers with deposition.^47,48^ The behavior of silver nanoparticles with the cells is the same as microorganisms, with some little differences physiologic in their membranes.^49^ The foams did not show cytotoxic behavior.^39−45^ The last authors reported no cytotoxicity of PU foams partially substituted with fibers *in natura*, showing no cytotoxicity to human fibroblasts. In this way, in our findings, the potential risk of the studied composite is low for the suggested application, and this material may be a potential application for industry.

## 4 Conclusions

The partial replacement of the PEG was found to be viable up to 25 and 35% for corn cob fiber foam and treated corn cob fiber foam, respectively. Foams with lower fiber content exhibited properties similar to those of foam without added fiber, making them suitable for upholstery, mattresses, sofas, etc. Intermediate foams, while losing resilience, acquired rigidity and had excellent applications for upholstery in general and pillows due to their retardation after compressive interaction. Foams with a high fiber content diverged from the properties of the original foam without added fiber but exhibited properties commonly found in insulation, acoustic, and thermal foam sectors. The chemical treatment of fibers proved effective with the use of a solution, resulting in a lower ash content compared to that of untreated foam.

Moreover, it was possible to increase the fiber content in the foam by 35% through treatment. However, it is worth noting that despite the higher fiber content in the foam fibers treated in this way did not reach commercial use due to their poor performance in the permanent compression test. Future research should consider changing the treatment concentration to improve these results. Although the addition of corn cob fibers resulted in reduced mechanical properties at higher fiber concentrations, the increment in the fiber content relies on an environmental appeal for future material. Foams with up to 15% fiber content maintained desirable viscoelastic properties, while higher fiber loadings proved to be suitable for thermal and acoustic insulation applications. This balance between performance and sustainability highlights the need for further optimization to improve both the mechanical properties and environmental benefits.

The process of depositing silver nanoparticles shows promise to produce foam with antimicrobial activity. Inhibition of microorganism growth was observed, and in some cases, they were eliminated. The absence of inhibition in the direct agar plate assay is a positive characteristic, as it indicates that the nanoparticle impregnated in the fiber has the potential to be nontoxic. Therefore, replacing the PEG reagent with corn cob fibers *in natura* and adding AgNPs are viable processes for foam production. This substitution and adding makes the process more cost-effective, allows the foam to degrade more rapidly in nature, reduces its environmental impact, and offers antimicrobial activity in the resulting product.
